# Cosmetic lengthening: what are the limits?

**DOI:** 10.1007/s11832-016-0791-z

**Published:** 2016-11-11

**Authors:** F. Guerreschi, H. Tsibidakis

**Affiliations:** Lecco Ilizarov Unit, A. Manzoni Hospital, Milan, Italy

**Keywords:** Short stature, Limb lengthening, Ilizarov hybrid circular external fixator, Cosmetic leg lengthening

## Abstract

**Objectives:**

In the last decades, limb lengthening has not been limited to the treatment of patients with dwarfism and deformities resulting from congenital anomalies, trauma, tumor and infections, but, has also been used for aesthetic reasons. Cosmetic lengthening by the Ilizarov method with circular external fixation has been applied to individuals with constitutional short stature who wish to be taller.

**Materials and methods:**

From January 1985 to December 2010, the medical records of 63 patients with constitutional short stature (36 M, 27F; 126 legs) who underwent cosmetic bilateral leg lengthening using a hybrid advanced fixator according to the Ilizarov method, were reviewed, retrospectively. The mean age was 24.8 years, while the mean preoperative height was 152.6 cm. Paley’s criteria were used to evaluate problems, obstacles, and complications from the time of surgery until 1 year after frame’s removal.

**Result:**

The mean lengthening achieved in all patients was 7.2 cm (range: 5–11 cm), with a mean duration of treatment of 9 months and 15 days (range: 7–18 months). The mean follow-up time was 6.14 years (range 1–10).

**Conclusion:**

The cosmetic leg lengthening was helpful to all patients, improving their social capabilities and self-confidence. All patients considered their stature as normal and they reported satisfaction and gratification with important changes in their professional and personal life. Cosmetic leg lengthening may raise some ethical objections and for that reason patients should be well informed about all the risks and complications related to this type of surgery.

## Introduction

Over the past several decades, bone lengthening has been performed not only for the treatment of dwarfism and/or skeletal deformities caused by congenital abnormalities, trauma, tumor or infections but also for aesthetic reasons [1, 2].

Cosmetic lengthening using the Ilizarov method with circular external fixation has been applied to individuals with constitutional short stature who wish to be taller. This new application is called cosmetic leg lengthening or symmetrical extended limb lengthening, and has been compared with the simplest options of plastic surgery [3, 4].

However, in the literature little is known about the use of the Ilizarov method to gain height for aesthetic/cosmetic reasons, and the correlated inherent risks and benefits of this type of surgery.

The aim of this work was to present our experience at the Ilizarov Unit of the A. Manzoni Hospital of Lecco (Italy) for cosmetic bilateral leg lengthening using an hybrid external circular frame according to the Ilizarov technique, and to present the limits of this procedure.

## Patient inclusion criteria

All patients were evaluated by an expert medical team who examined the impact of short stature on the patient’s everyday life, and how they might cope with difficulties encountered during treatment. Actual and perceived problems related to short stature were also taken into account. Difficulties in daily work and life, driving motorcycles or large bicycles were also considered. Functional limitations of short stature were considered a valid and good motivation for gaining height surgically. Patients requested surgery particularly for professional reasons, such as military or police career, models, and business people who felt uncomfortable in meetings etc. due to their short stature.

Regarding height distribution, the normal bell curve was considered, and patients were divided as shown in Table 1. Normal height was considered ±3 standard deviations (SD) from the mean. A stature below 3SD in patients without dwarfism and/or skeletal deformities was considered as a constitutional short stature. The lower limit of normal stature for Caucasian people was 5′5″ (166 cm) for males and 5′0″ (153 cm) for females.

**Table 1:** Values of normal stature (*SD* standard deviation)

Height (cm)		Percentile	SD
Women	Men		
174	189	95	+3
171	185	90	+2
167	181	75	+1
163	176	50	Mean
160	172.5	25	−1
156	169	10	−2
153	166	5	−3

All patients who underwent cosmetic leg lengthening were under the 5th percentile for age and gender, and without any dwarfism and/or skeletal deformities or hormonal deficiencies. A detailed history of all previous aesthetic interventions was included to exclude dysmorphophobia [5–7].

Psychological evaluation of all patients and their families, anthropometrical measurements with particular attention to the proportions of the limbs and trunk, and radiological examination for deformity and/or leg length discrepancy were performed. Patients were also informed about the duration of treatment and all the possible complications during surgery and after removal of the frame. Informed consent was obtained from all patients. Patients who accepted to undergo surgery were also invited to discuss their treatment with at least two other patients before and after surgery.

In cases of deformity and leg discrepancy, simultaneous correction was also obtained.

## Patients and methods

The study was performed according to the ethical standards of the Declaration of Helsinki (1964) and its later amendments. From January 1985 to December 2010 the medical records of 63 patients with constitutional short stature (36 males, 27 females for a total of 126 legs) who underwent cosmetic bilateral leg lengthening using a hybrid advanced fixator according to the Ilizarov method, were reviewed retrospectively [4, 8, 9]. The mean age was 24.8 years (range 17–48 years; 27.8 for males and 22.9 for females) while the mean preoperative height was 152.6 cm (range 140–172 cm; 154.4 cm for males and 145.2 cm for females). Eight patients also had varus knee deformity which required correction during treatment. All patients practiced non-competitive sports.

Preoperative clinical/radiographic evaluation and surgical planning were performed for all patients.

Paley’s criteria were used to evaluate complications for this procedure, including postoperative assessment of all problems, obstacles and complications from the time of surgery until 1 year after removal of the frame [8].

Problems were defined as any potential difficulties arising during the treatment period and fully resolved by the end of the process by non-operative means. Pin track infection, docking drift, wound breakdown, and delayed consolidation were included in this category.

Obstacles were defined as any potential difficulties arising during the treatment period and fully resolved by the end of the process by operative means. Non-union, joint contracture, atrophic or fracture through regenerated bone, axial deviation, leg length discrepancy, equinus, and early fibular consolidation were included in this category.

Complications were defined as any local or systemic complication (intraoperative/postoperative) or difficulty found during the stretching or stabilization that remained unresolved until the end of the treatment period, and any early or late difficulty observed after treatment. Persistent knee contraction, amputation due to non-union/poor regenerate bone or persistent infection, reflex sympathetic dystrophy and neurological disturbances were included in this category [10].

Patient follow-up was performed every 3 months for the first year and then every 2 years, evaluating patient satisfaction, possible axial deviation, range of movement of the knee and ankle, pronation of the foot, leg length discrepancy and scars. According to the patient and physician scores based on these parameters, the outcome of surgery was classified as poor (0–4), fair (5–9), good (10–14) or excellent (15–18).

Finally, psychological outcome after treatment was evaluated by determining improvement in self-esteem, distress, shyness and quality of life. All patients were asked if they would undergo surgery again and whether they would recommend it to others of similar stature.

## Operative technique

The hybrid advanced fixator is a modification of the classic Ilizarov fixator [9] combining Kirschner wires with half-pins and full rings with arches [1, 11]. The standard apparatus (3 rings and one half-ring for the leg) was assembled preoperatively with the rings being sized directly onto the patient’s legs and the wires and half-pins applied with routine transfixation. The whole construct was connected with threaded rods [11, 12].

Two osteotomies, using the Gigli saw or multiple drill holes were carried out—one below the tibial tuberosity and the other at the supramalleolar level. A fibular osteotomy was performed at the junction of the middle and distal third of the leg. A hand-controlled drill with a speed of 0–1000 revolutions/min was used for the insertion of the wires, and pilot holes were drilled before insertion of the half-pins.

Lengthening was started 10 days after surgery at a rate of 0.75 mm per day (one-quarter turn every 8 h) for each tibia osteotomy.

Weight-bearing was encouraged on the second day after surgery, according to tolerance, followed by a rehabilitation program of gradual increased load-bearing and physiotherapy.

Pin care began the day after surgery using hydrogen peroxide and betadine.

Patients were discharged with instructions for bi-weekly care of the pin site. Clinical and radiological examinations were carried out every 30–40 days to assess new bone formation, pin sites, patient satisfaction, tibia length and joint movements.

Bilateral leg lengthening of 5–8 cm was considered satisfactory. Radiological criteria for successful lengthening included complete bone bridging in at least two radiographic projections. Bone regeneration was assessed clinically by loosening the connecting rods and applying stress. When consolidation of new bone was confirmed clinically and radiologically, the frames were removed under sedation. A fiberglass cast or braces including the foot were applied for a mean of 6 weeks.

## Results

The mean lengthening achieved in all patients who underwent surgery was 7.2 cm (range 5–11 cm), with a mean duration of treatment of 9 months and 15 days (range 7–18 months).

**Fig. 1:**
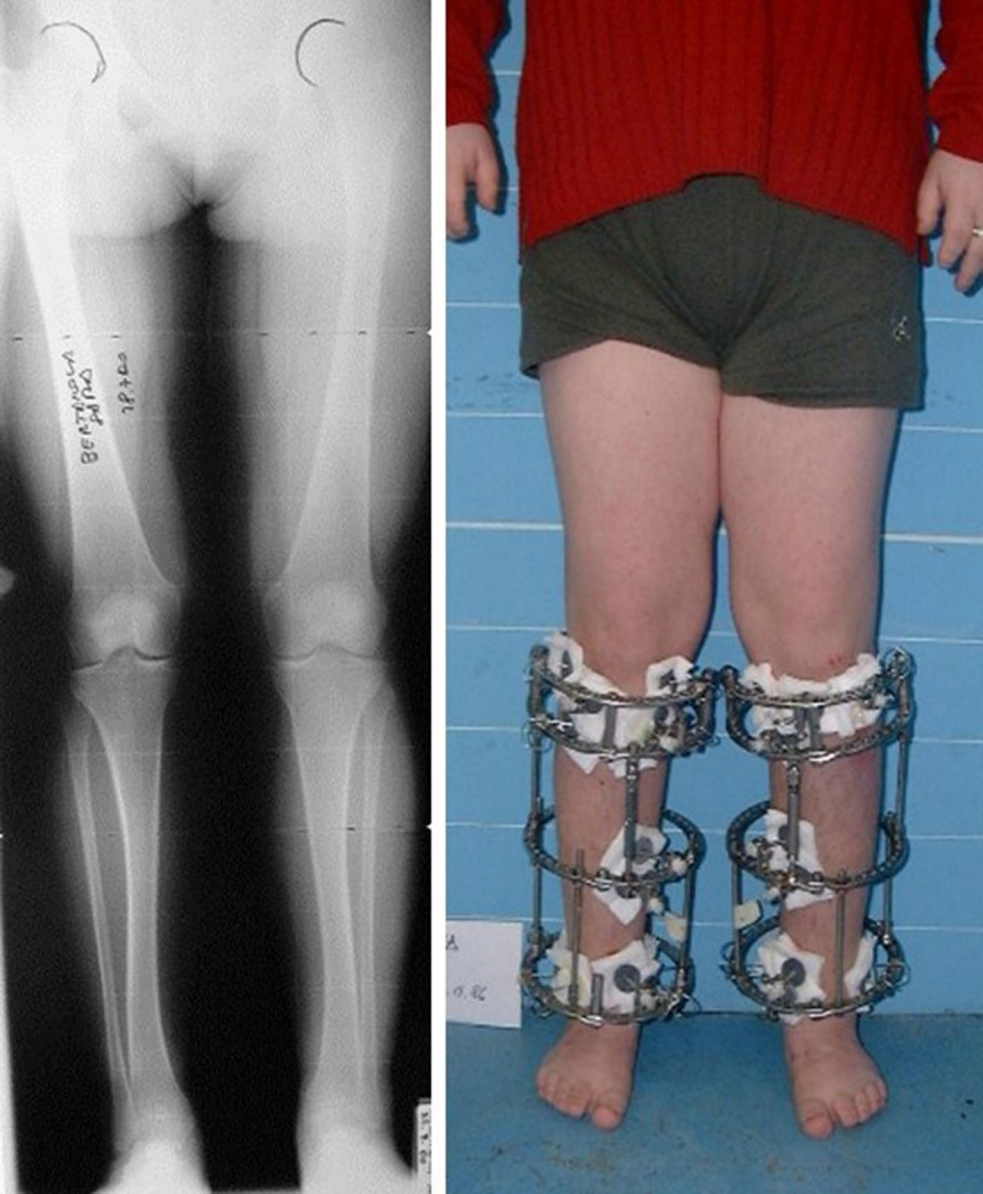
X-rays before treatment in a 17-year-old patient, SD −2

After removal of the frame, the fiberglass cast was applied in 31 patients (49.2%) and braces including the foot in 21 patients (33.3%). All patients performed physiotherapy for a mean of 6 weeks (range 4–8 weeks). The mean follow-up time was 6.14 years (range 1–10). Varus knee deformity was corrected simultaneously in 8 patients. In 21 patients (33.3%), bilateral lengthening of the Achilles tendon was also necessary to correct the equinus deformity that developed during distraction (Fig. 1).

According to Paley’s criteria, 102 difficulties were observed—42 problems, 54 obstacles, and 6 complications (Table 2).

**Table 2:** Difficulties according to Paley’s criteria [8]

	Number	Treatment
Difficulties	102/126	
1. Problems	42/126	
Pin trac infection	25	Oral antibiotics
Proximal tibia procurvatus	4	On-going correction
Proximal tibia recurvatus	2	On-going correction
Distal tibia varus	2	On-going correction
Distal tibia valgus	5	On-going correction
Limited ankle dorsal flexion	4	On-going correction
2. Obstacles	54/126	
Athrophic new bone	2	Bone graft
Equinus foot	42	Achilles tendon lengthening
Collapse of new bone	5	Ilizarov apparatus reapplication
Leg length discrepancy	1	Ilizarov apparatus reapplication
Early fibular consolidation	4	Second fibular osteotomy
3. Complications	6/126	
Foot pronation	6	3 cases of subtalar joint fusion

Regarding pin tract infection, we reported 12 grade 1 (pain, erythema, or tenderness around the pin site), 8 grade 2 (characteristics of grade 1 infections plus serous drainage) and 5 grade 3 (characteristics of grade 1 infections plus purulent drainage) according to Gordon’s grading system [13]. Pin-site infections were treated by oral antibiotics (amoxicillin) [14, 15] except for one, which required intravenous antibiotics and one half-pin removal without compromising the frame’s stability. No cases of radiographic osteolytic changes at the pin site (grade 4) or ring sequestrum/osteomyelitis (grade 5) were reported.

Two cases of atrophy of the new bone formation at the distal distraction were treated with autologous cancellous bone grafting from the iliac crest.

Due to early bone consolidation, a revision of the fibular osteotomy was necessary in four limbs.

In five limbs, collapse of the regenerate bone was observed after removal of the frame—a proximal varus deformity occurred in two, and a proximal anterior bowing with distal valgus deformity occurred in the other three. Application of a new Ilizarov frame was performed until complete bone healing and correction were obtained.

Hinges were applied for correction of all axial deviations observed such as a proximal anterior tibial bowing (4° and 5°) resulting in a minor loss of knee extension (4 limbs), a slight recurvatum of the proximal tibia of 3° which did not affect the movement of the knee (2 limbs), a varus of the distal part of the tibia of 4° (2 limbs), a valgus of the distal part of the tibia (3°–5°) resulting in pronation and minor stiffness of the subtalar joint (5 limbs), and a limitation of the ankle dorsiflexion of 20° (4 limbs). A leg length discrepancy of 10 mm was observed in only one case and a new external fixator was applied.

Foot pronation was observed in six limbs and a subtalar joint fusion was necessary to stabilize the foot in three cases.

No fat embolism, deep vein thrombosis, or pulmonary embolism was observed during the entire follow-up period.

**Fig. 2:**
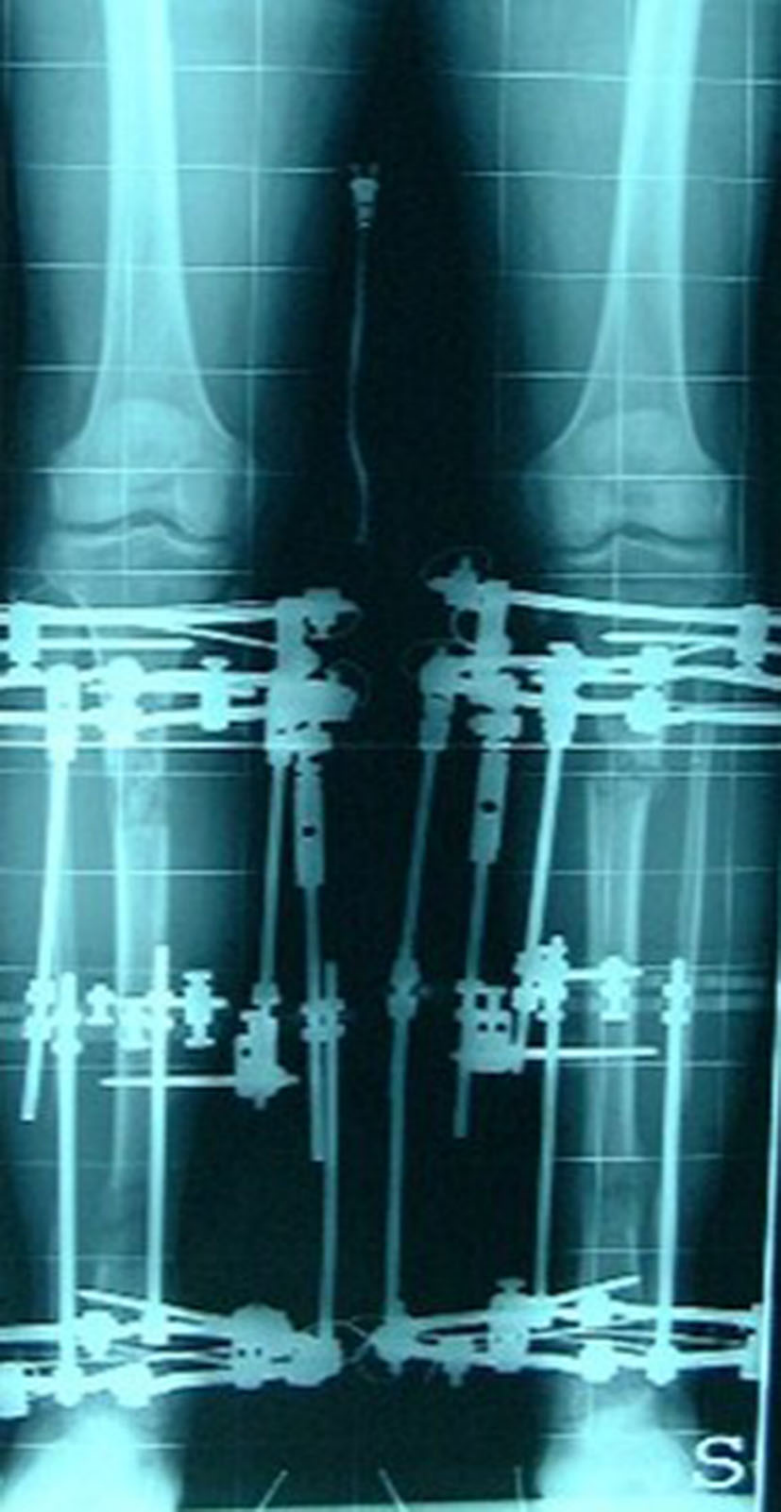
Two-level lengthening, start of treatment

**Fig. 3:**
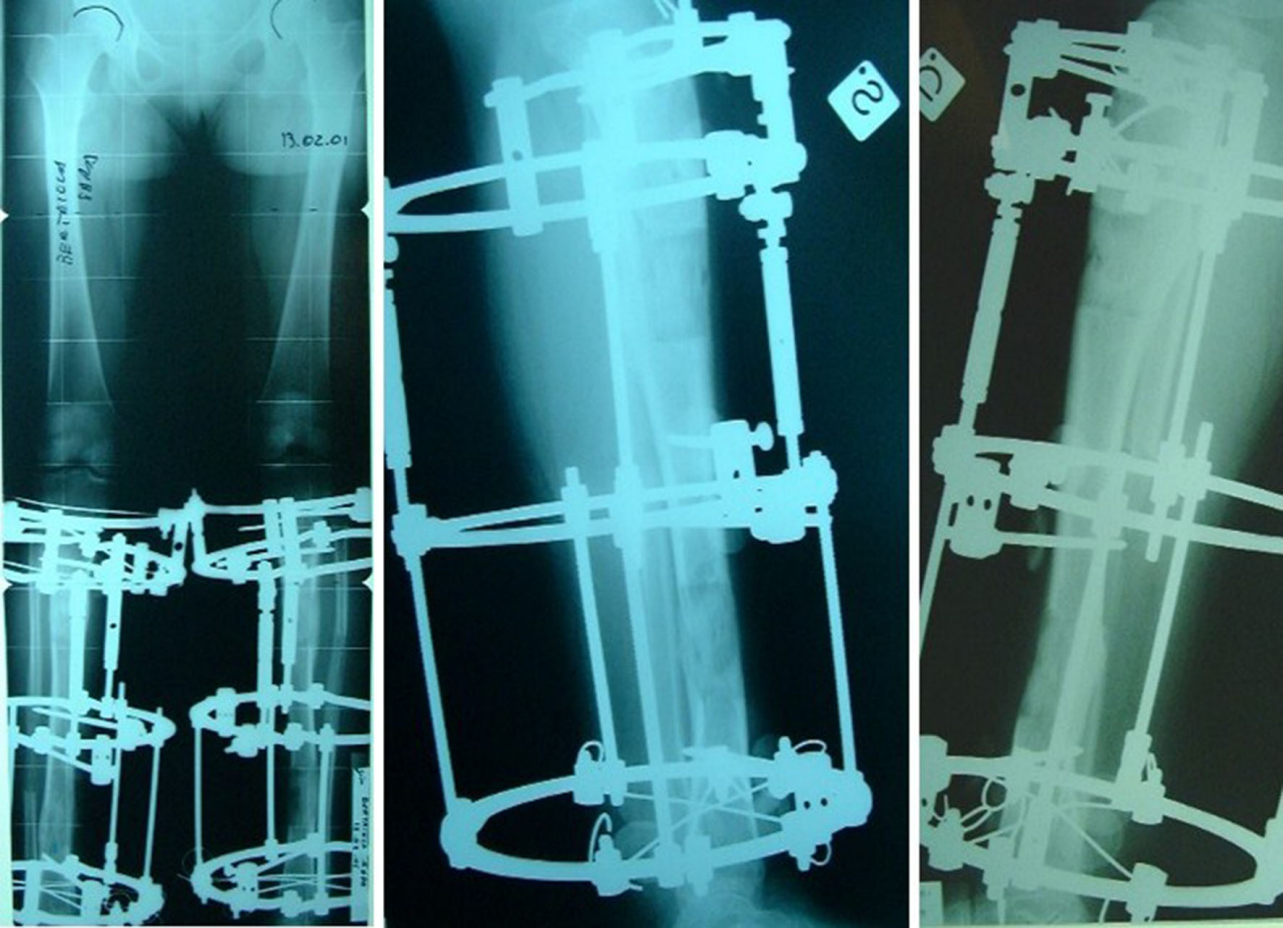
X-rays before frame removal, with hinges for axial valgus deviation correction

**Fig. 4:**
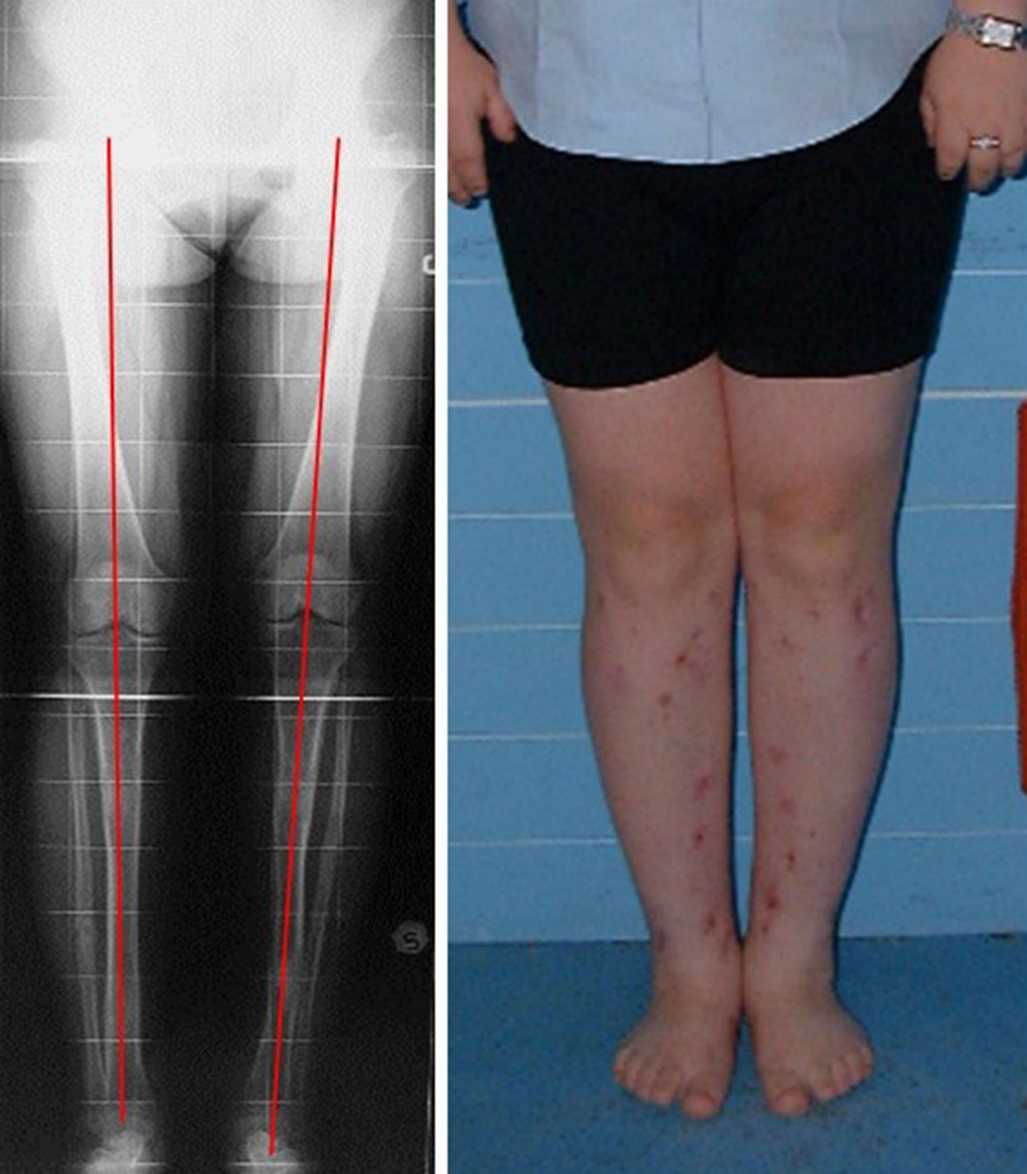
X-ray after frame removal and photograph of patient

**Fig. 5:**
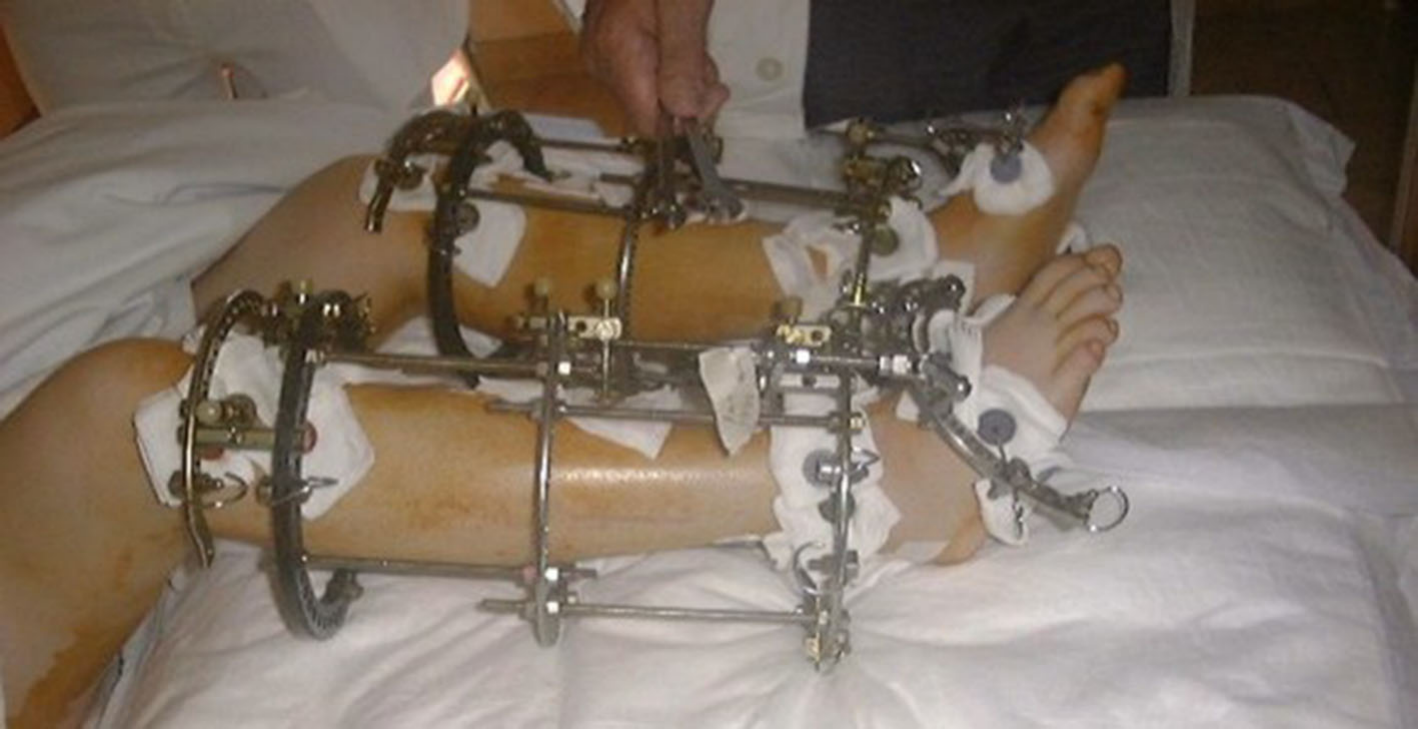
Achilles tendon lengthening and joint contracture correction

At the latest follow-up, all patients were satisfied with improvements in self-esteem, distress or shyness and quality of life. They all stated that they would recommend the treatment to others of similar stature. When asked whether they would have this surgery again, 53 answered positively, and the remaining 10 were undecided (Figs. 2, 3, 4, 5).

Based on the parameters of patient satisfaction, axial deviation, restricted joint movement, pronation of the foot, leg length discrepancy and scars, the outcome was excellent in 56 patients (88.8%), good in 5 (7.9%) and fair in 2 (3.1%).

The final aesthetic effects were satisfactory in all cases and all patients continued with their previous sport activities.

## Discussion

In our study we surgically treated patients with constitutional short stature, defined as a height under the 5th percentile for age and gender, without any dwarfism and/or skeletal deformities and/or hormonal deficiencies [5, 6]. Although short stature is not considered as a disease, it can cause psychological [6, 7, 16] and functional disadvantages, and can have a radical influence on a person’s life [17, 18].

Patients with dysmorphophobia or body dysmorphic disorder are not suitable candidates for this type of cosmetic surgery. This disorder is a distressing and impairing preoccupation with an imagined or grossly exaggerated defect of appearance. It is associated with high rates of occupational and social disability, hospitalization and suicide attempts [19, 20]. Patients with dysmorphophobia usually seek cosmetic surgery to alter their subjective perceived abnormality. Psychological evaluation before surgery is mandatory to exclude such patients, even if some surgeons do not take this particular aspect into consideration and proceed with surgery [7]. A scrupulous preoperative psychological evaluation can help the surgeon to better understand the patient’s body perception and their expectation after surgical cosmetic lengthening.

Problems, obstacles, and complications of limb lengthening using the Ilizarov technique represent another limit for this type of surgery. According to our results, the use of the Ilizarov frame for cosmetic limb lengthening is a technique without major complications. However, it requires careful follow-up and should be performed by orthopedic surgeons who are familiar with the circular frame and experienced in limb lengthening and deformity correction. Patients who are candidates for cosmetic orthopedic surgery should be carefully selected as their co-operation is necessary for a successful clinical outcome [17].

Pin site scars at the end of treatment represent another important limitation. In our study, the majority of patients reported dissatisfaction with residual skin scars, without any impact on their social life.

Finally, any type of cosmetic surgery is not refunded by any medical insurance or public health system, and this may be a major limiting factor for patients who seek cosmetic stature lengthening.

The cosmetic leg lengthening was helpful to all patients, improving their social capabilities and self-confidence, as reported at the latest follow-up visit. All patients considered their stature as normal and they reported satisfaction and gratification with important changes in their professional and personal life.

Cosmetic leg lengthening may raise some ethical objections and for that reason patients should be well informed about all the risks and complications related to this type of surgery.

It is the opinion of the authors that the Ilizarov method for cosmetic limb lengthening is a valid and good technique without major complications. However, it requires careful psychological evaluation, and patients should be highly motivated, fully informed and understand this type of surgery and its possible complications.
